# Passive flow control via tip grooving and stall fencing mechanisms of a marine energy harvesting turbine

**DOI:** 10.1038/s41598-023-28300-0

**Published:** 2023-02-15

**Authors:** Tapas K. Das, Nazrul Islam, Abdus Samad, Amjad Ali Pasha

**Affiliations:** 1 Department of Mechanical Engineering, King Abdulaziz University, Jeddah, 21589 Saudi Arabia; 2 Wave Energy and Fluids Engineering Laboratory, Department of Ocean Engineering, Indian Institute of Technology Madras, Chennai, 600036 India; 3 Aerospace Engineering Department, King Abdulaziz University, Jeddah, 21589 Saudi Arabia

**Keywords:** Ocean sciences, Energy science and technology, Engineering

## Abstract

Remarkable advancement in wave energy conversion technology has taken place in recent years. Due to its simplicity, the Wells turbine has been one of the most widely used power take-off mechanisms in an oscillating water column type wave-energy conversion device. However, the turbine suffers from several challenges due to its narrow operating range, which hinders the commercial feasibility of the system. Several aerodynamic applications have successfully used passive control methods to modify the flow conditions. This work applied a combination of stall fences and casing grooves for passive flow control of a Wells turbine. The computational fluid dynamics (CFD) technique is used to analyze the modified turbine numerically. The casing groove modified the tip-leakage vortices, interacted with local vortices created by the stall fences, and helped reattach the flow at higher flow coefficients. As a result, the modified turbine increases the operating range up to 33.3%. In addition, the peak-to-average (PTA) power ratio decreased by up to 27.7%.

## Introduction

As global energy consumption increases gradually, governments and policymakers promote new schemes to explore alternative renewable energy sources. The International Renewable Energy Agency reported a possible rise of the global renewable energy share from 25% in 2017 to 85% by 2050^1^. As of 2020, the renewable energy share of total electricity capacity is approximately 37%^2^. Among the different renewable energy sources available, the technologies to convert hydro, wind, and solar energy are the most advanced and make up almost 99% of renewable energy consumption^2^. However, unconventional renewable energy sources such as geothermal, biomass, and marine energy have gained interest. The current installed capacity of marine renewable energy is approximately 527 MW. Marine renewable energy is generally composed of tidal energy, marine current energy, and wave energy. Countries with long coastal lines can meet their energy demand by harnessing and converting this wave energy into electricity.

The countries in the Gulf region rely heavily on fossil fuels for their energy demand. For example, it is estimated that the electricity demand in Saudi Arabia will exceed 120 GWh by 2032^3^. One of the reasons behind paying more attention to renewable energy is to reduce CO_2_ emissions caused by relying mainly on fossil fuels. For example, CO_2_ emissions in Saudi Arabia have rapidly grown from 252,000 Gg in 2000 to 446,000 Gg recently^4^. Therefore, more attention has been directed toward a viable renewable energy resource. A numerical assessment of wave energy potential has been carried out for the Red Sea (i.e., the west coast of Saudi Arabia) using the wave conditions of the period between 1979 and 2010 by Aboobacker et al.^5^. The amount of wave power potential discovered in the deep waters of the Red Sea ranges up to 4.5 kW/m, while it ranges between 0.66 and 1.16 kW/m near the central coast.

**Figure 1:**
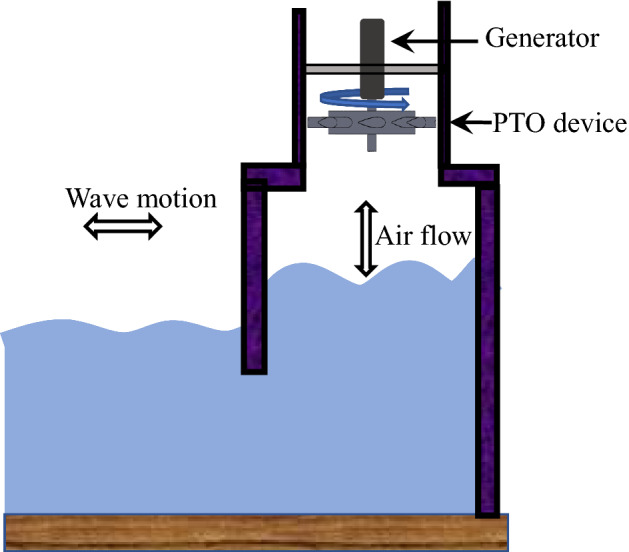
An oscillating water column (OWC) with a power take-off (PTO) device.

For several reasons, it’s challenging to convert wave energy into useful electrical energy. These challenges are the harsh ocean environment, the unpredictability of the wave climate, and the conversion technology to be implemented. Past researchers have developed several different wave energy conversion technologies based on distance from the shoreline, direction of the incoming wave, and water depth. Various concepts such as a combined wind and wave energy technology^6^ or a fully enclosed inertial body concept^7^ have shown promising results. For wave energy technologies, the oscillating water column (OWC) proved to be an efficient wave energy converter with several prototypes developed and tested in actual sea conditions^8–10^. The OWCs can be built near the shoreline or offshore locations, depending on the wave condition. The device consists of a chamber-like structure, partially submerged in water with an opening in both the top and bottom (Fig. 1). The opening at the bottom allows the ocean waves to enter the chamber and create an oscillating airflow due to the change in air pressure. The opening at the top is connected to a duct that houses a turbine as a power take-off (PTO) device. The change in pressure inside the oscillating water column chamber causes the turbine to rotate, and a generator is used to produce electricity from the turbine.

**Figure 2:**
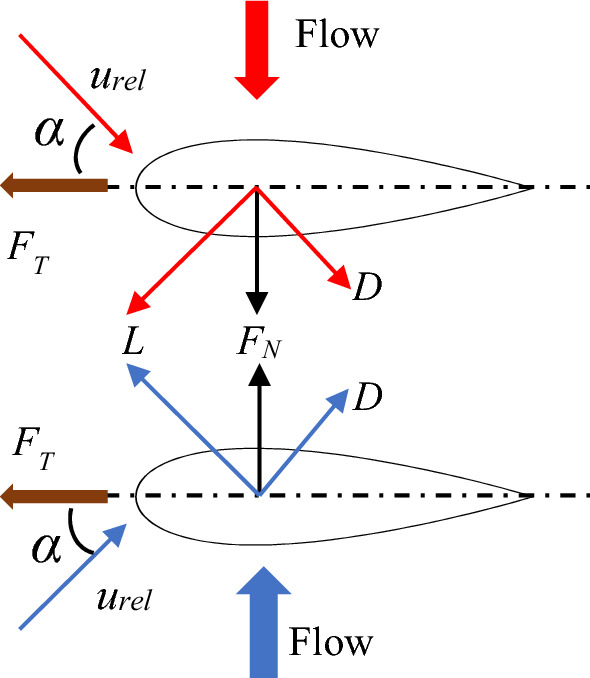
Forces and velocities on Wells turbine blade.

The OWC turbine must be a bidirectional turbine that can revolve in one direction regardless of the chamber's bidirectional airflow. The Wells turbine is one such type of turbine that is popular among OWCs due to its ease of design and installation. The turbine's symmetric blades rotate in one direction even when the airflow direction changes with each trough and crest of the waves^11^. The stall phenomenon—a difficulty associated with aerofoils subjected to a high angle of attack (AoA)—is one of the principal downsides of the turbine, despite its many benefits. In an OWC, the turbine blades are subjected to a relative velocity which consists of the inlet velocity and rotational speed (as shown in Eq. 1 and Fig. 2). With the turbine rotational speed fixed, an increase in inlet velocity increases the AoA of the relative velocity. After a certain AoA, the stall phenomenon sets in, drastically reducing the tangential force (and hence the torque) developed by the turbine. As a result, the maximum inlet velocity or rotational speed that the turbine can operate is limited by the AoA at which the stall occurs.

1a $$F_{T} = L\sin \alpha - D\cos \alpha$$

1b $$F_{N} = L\cos \alpha + D\sin \alpha$$

where *F*_*T*_ is the tangential force, *F*_*N*_ is the normal force, *L* is the lift force, *D* is the drag force and *α* is the angle of attack.

Past researchers have made several efforts to increase the AoA and delay the onset of the stall in the Wells turbine. Direct blade geometry modifications such as a change in blade profile^12^, non-uniform solidity distribution^13^, blade sweep^14,15^, blade skew strategy^16^, and 3-dimensional blade^17^ showed significant improvement in turbine performance. In addition, the use of a casing groove has demonstrated tremendous potential for stall delay by reducing the tip leakage losses^18^. Past researchers also applied varieties of optimization methods for better performance and delaying stall of the turbine^19–21^. The modified blade geometry considerably enhanced the turbine's performance.

The Wells turbine blades operate in the same way that an aerofoil does. In aerofoil, passive flow control techniques aid in changing the flow field around the turbine blade. When applied to aerofoils, various passive flow control techniques, such as plasma actuators, blowing and suction, vortex generators, and riblets, have demonstrated the potential for stall delay and drag force reduction. Table 1 summarises the Well turbine's various passive flow control techniques and the resulting improvement. As can be seen, all of these passive flow control techniques significantly improved turbine performance. On the other hand, a combination of different passive flow control can substantially improve turbine performance even further than previously achieved.

**Table 1:** Wells turbine modification using passive flow control.

A passive flow control method	Outcome
Single suction slot^22^	Torque coefficient increased by 40%, operating range by 17%
Multiple suction slots^23^	Torque coefficient increased by 26.7%, operating range by 51%
Undulating leading edge^24^	Operating range increased by 12.5%
Gurney flap^25^	The torque coefficient increased by 10.7%
Static extended trailing edge^26^	Power output increased by 97%, Operating range increased by 22%
Endplate^27^	Peak efficiency increased by 16%
Stall fence (SF)^28^	The operating range increased by 16.7%

Stall fences (SF) have been demonstrated in a previous study to extend the operational range of a Wells turbine but at the expense of the turbine's peak torque. The use of a casing groove (CG) has, on the other hand, boosted peak turbine torque. The current research aims to integrate the effects of SFs with CG to create a better Wells turbine. Numerical analysis was conducted with both the stall fence and the casing groove. Different CG depths are investigated through numerical simulation, and the results have been compared to the reference turbine. A detailed analysis of flow is then performed to understand better the flow pattern around the blade profile caused by the presence of SF and how CG influences the tip leakage flow.

## Geometric features

**Figure 3:**
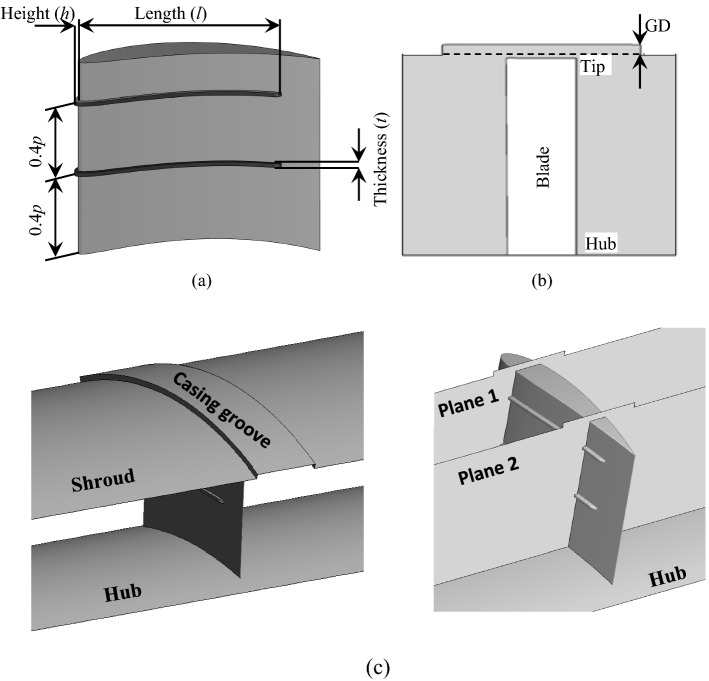
Geometric configurations of the blade with stall fences and casing groove (**a**) details of SFs and (**b**) details of CG (**c**) details of the combined stall fence and casing groove.

Generally, the Wells turbine blades have a symmetric NACA profile. The turbine considered here has eight blades made up of the NACA0015 profile. The blade's chord length (*C*) is 125 mm, and the turbine solidity is 0.64. The hub radius of the turbine is 0.2 m, and the hub to tip ratio is 0.67. The clearance at the tip is 1% *C*. As shown in Fig. 3a, the blades are associated with two SFs. The two stall fences are separated by 40% of the blade span (*p*) from the hub to the tip direction. The optimum geometric dimensions of the SFs are decided based on the optimization carried out in^28^. The length of the fences is 80% *C*, and the height and the thickness of the blade are 1.6% *C*. Based on the casing treatment scheme explained in^18^, a CG is added to the blade casing in addition to the fixed tip clearance, as shown in Fig. 3b. Figure 3c shows a detailed three-dimensional view of the blade with the stall fence and casing groove. Two planes (plane 1 & plane 2) are used to show a cross-sectional view of the blade. The groove depth (GD) is defined as a percentage of *C* and varied to different depths to investigate the impact of tip leakage losses on turbine performance.

## Numerical solution approach

**Figure 4:**
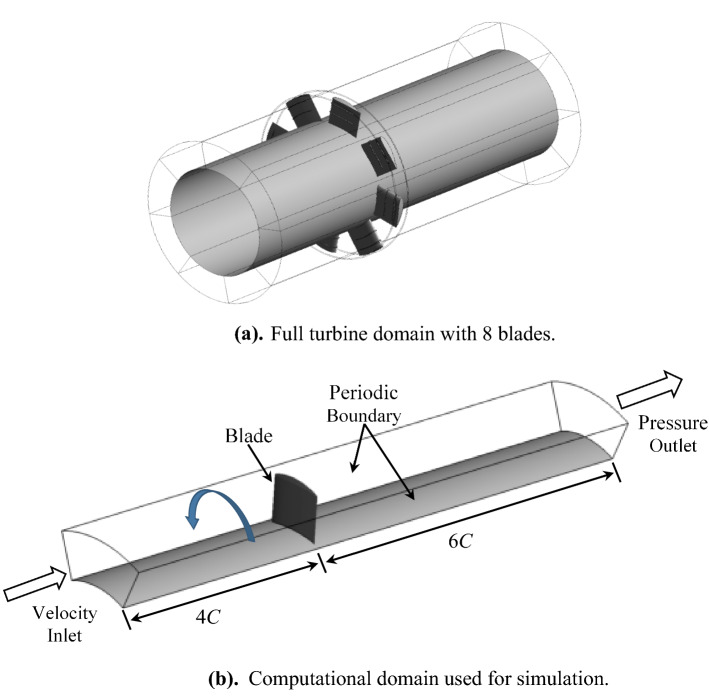
(**a**) Full turbine domain with 8 blades. (**b**) Computational domain used for simulation.

The numerical simulation was performed in ANSYS CFX 16.1- a commercial CFD solver customized for turbomachinery applications. The full geometry of the turbine consists of 8 blades on a circular hub inside a duct or casing (Fig. 4a). The numerical simulation's computational domain is made up of one blade, and a 45° sector of the duct, with periodic boundary conditions applied on both meridional sides, as shown in Fig. 4b. The inlet of the domain has been four times the chord length, and that domain’s outlet is six times the chord length. The velocity inlet boundary condition is employed at the domain's inlet. At the domain's outlet, the pressure outlet boundary condition is utilized. As indicated in Fig. 4b, the turbine operates at a constant speed of 2000 rpm.

**Figure 5:**
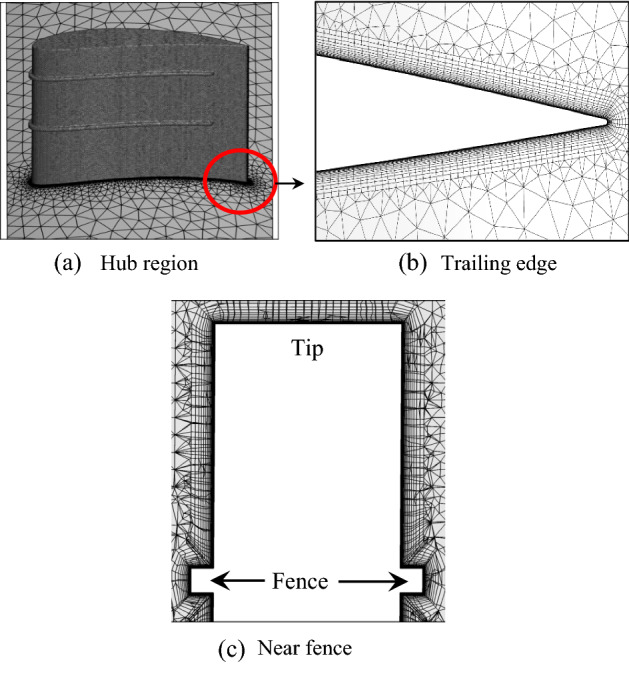
Discretization of the computational domain around the blade.

The computational domain is discretized into an unstructured tetrahedral mesh (Fig. 5). However, for accurate capture of the flow behavior around the blade, the boundary layer around the blade is discretized with 20 prism layers. Based on the criteria of *y* +  < 1, the height of the first layer of the prism is kept at 11 µm. A growth ratio of 1.2 was selected for the height of the successive prism layers.

The turbines were numerically examined using the CFD solver to obtain the solution of the Reynolds Averaged Navier–Stokes (RANS) equations. The numerical analysis was conducted for a constant incompressible flow. For brevity, more information about the solver can be found in^29^, which is not provided here. Because the simulation resembles flow over an aerofoil surface, it is critical to capture the boundary layer phenomenon to obtain a good numerical solution. The *k*-*ω* SST model combines the *k*-*ω* models applied to near-wall flow and *k*-*ε* models to far-field freestream flow. For the flow conditions around the aerofoil considered here, it’s wise to choose the *k*-*ω* SST model. Previous numerical investigations on symmetrical NACA profiles have revealed that the *k*-*ω* SST model successfully captured the boundary layer and forecasted the stall point better than other models^30,31^. Using the *k*-*ω* SST model, previous computational investigations on the Wells turbine also accorded experimental data^18,32^. As a result, the numerical analysis is also performed using the *k*-*ω* SST model.

**Figure 6:**
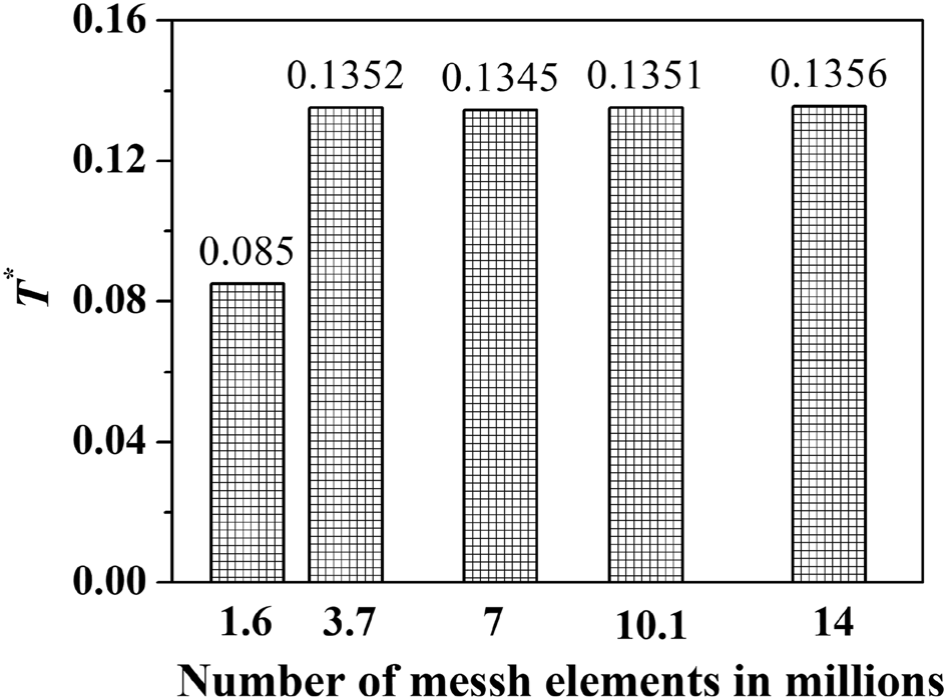
Variation of torque coefficient with grid numbers at *φ* = 0.225.

The number of elements in the computational domain was varied by changing the mesh scale. Five different meshes were generated to check the mesh's dependence on the solution. Figure 6 shows the grid impendence study at a flow coefficient of 0.225. The mesh count ranged from 1.6 million (coarse) to 14 million (fine). After a mesh size of 3.7 million, the torque coefficient value does not differ significantly. So further simulations were carried out using this mesh specification.

## Results and discussion

In the first step, the reference turbine's numerical model results are compared and validated with experimental results^33^. The turbine performance is measured by the torque produced and its efficiency. The turbine output parameters are specified in non-dimensional quantity as follows:

i. Non-dimensional pressure drop:
  2 $$\Delta P_{0}^{*} = \frac{{\Delta P_{0} }}{{\rho \omega_{r}^{2} R^{2} }}$$
ii. Non-dimensional torque:
  3 $$T^{*} = \frac{T}{{\rho \omega_{r}^{2} R^{5} }}$$
iii. Efficiency:
  4 $$\eta = \frac{power\;otput\;of\;the\;turbine}{{available\;power\;at\;the\;inlet}} = \frac{{T\omega_{r} }}{{\Delta P_{0} Q}}$$
iv. Flow coefficient:
  5 $$\varphi = \frac{{u_{a} }}{{r_{t} \omega_{r} }}$$
  where *T* is the total torque developed by the turbine and $\Delta {P}_{o}$ is the pressure difference between inlet and outlet of the turbine. *Q*, *u*_*a*_, *r*_*t*_, *ω*_*r*_ and *ρ* are the volume flow rate, inlet velocity, tip radius, rotational speed and density of air respectively.

**Figure 7:**
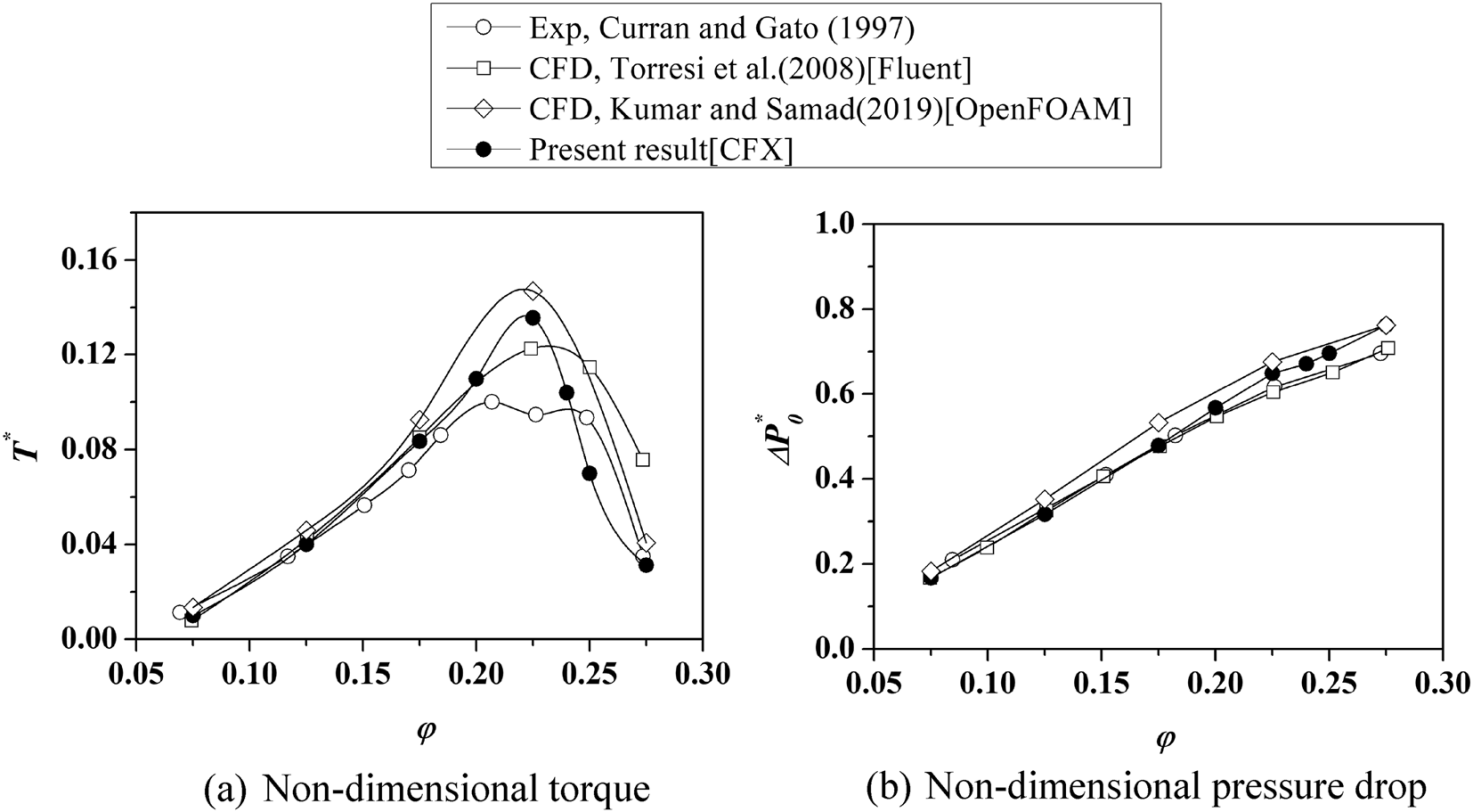
Numerical model validation using existing data.

Initially, the reference turbine is simulated at various inlet velocities similar to those used in the reference turbine experiment. Then, the non-dimensional turbine parameters are plotted against the flow coefficient. The speed of rotation of the turbine is fixed at 2000 rpm. By varying the inlet velocities, the flow coefficient can be changed. At eight different flow coefficients (0.075 ≤ *φ* ≤ 0.275 for 4.7 ≤ *u*_*a*_ ≤ 17.3 (in m/s)), the turbine is simulated. The current study's findings are compared to existing computational and experimental results, as shown in Fig. 7. The non-dimensional torque of the reference turbine is seen to be in good agreement with the experimental results until *φ* = 0.175. The maximum deviation from experimental values is limited to 5% in this region. In the case of the reference turbine, the stall begins near *φ* = 0.2. When compared to experimental results, numerical results overpredict torque coefficient values from this point to the point of deep stall (*φ* = 0.225). To better understand the deviation of the non-dimensional torque above *φ* = 0.2, the current numerical results are compared to previous CFD results of the same reference turbine by different authors^25,34^. The current predictions agree well with previous CFD results; they all overpredict the experimental non-dimensional torque value in the range *φ* = 0.2 to *φ* = 0.225. Simultaneously, the numerical values of the non-dimensional pressure drop agree well with the experimental measurements.

**Figure 8:**
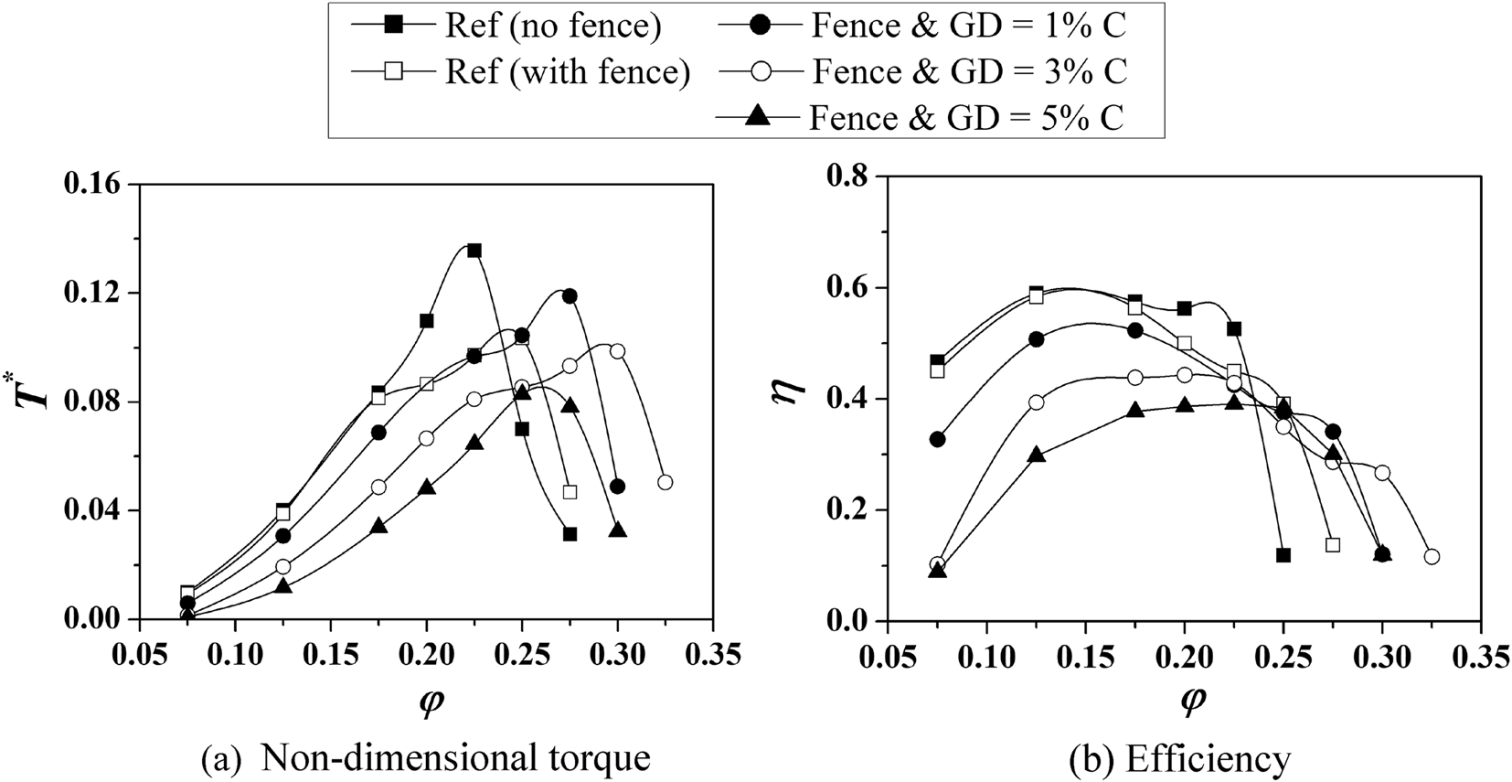
Comparison of different cases of the combined SF-CG case with two reference geometries.

The validated numerical model is used to simulate the new turbine blade geometries consisting of SFs and a CG over the blade tip. Three different case studies are performed with different CG depths (GD = 1, 3, and 5% *C*). Figure 8 compares different cases of the combined SF-CG case with two reference turbines: (1) with no SF or CG and (2) with only SF but no CG^28^. The comparison of the results shows two significant observations: (a) the turbine blade with a combined SF-CG case has a lower non-dimensional torque than the original turbine without any modification, and (b) the modified turbine has a wider operating range (i.e., delayed stall) compared to the original turbine. For the turbine with a combined SF and CG with GD = 1% *C*, the stall point is found to shift to *φ* = 0.275, whereas for GD = 3% *C*, the stall point is seen to shift further to a *φ* = 0.3. The operating range is wider than the reference case when only SFs are used without any CG. As the non-dimensional torque is reduced for the modified turbines, the efficiency also gets reduced compared to the original turbine. However, as the stall is postponed for the modified turbine, the efficiency drops more gradually than the sudden drop in the reference turbine, as shown in Fig. 8.

**Figure 9:**
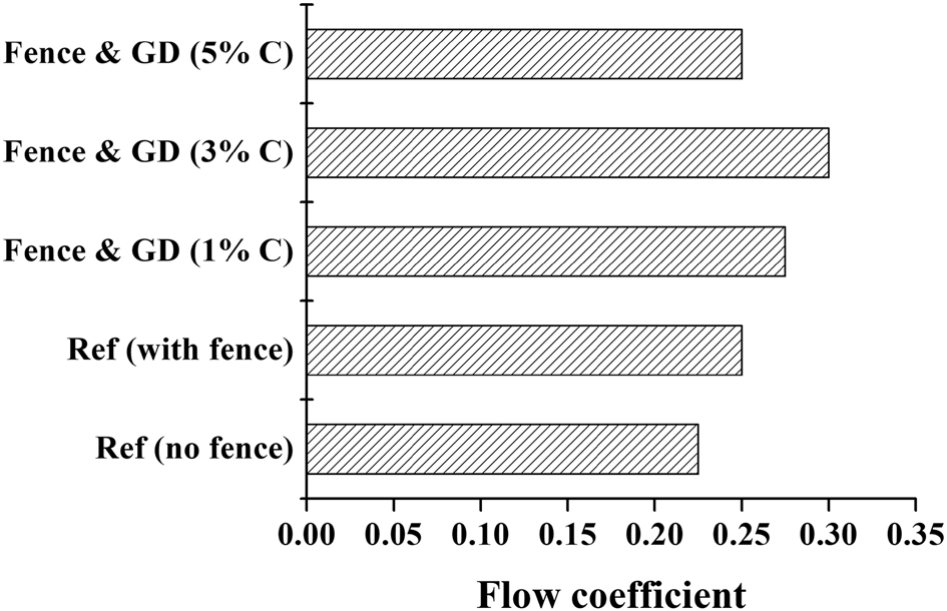
Comparison of stall points for different configurations.

Figure 9 shows the stall points for different combinations studied here compared to the reference turbines. The stall point of the turbine improves with the addition of a combined stall fence and casing groove. Additionally, for the designs with a combined stall fence and casing groove, the stall point improves gradually by increasing the groove depth from 1 to 3%. However, further increasing the groove depth increases the tip leakage flow, thus reducing the turbine's stall point or operating range.

One of the problems in the Wells turbine because of the stall phenomenon is the PTA power ratio, i.e., the operating region of the turbine is quite narrow and can generate torque only for a narrow band of inlet velocity conditions. To operate the turbine smoothly for a wider band of inlet conditions, one way to reduce the PTA power ratio. When the turbine operates at a fixed rotation speed, the turbine's power output is directly proportional to the torque. The combined SF-CG modification reduces the PTA power ratio of the turbine by up to 27.7%.

### Internal flow analysis

**Figure 10:**
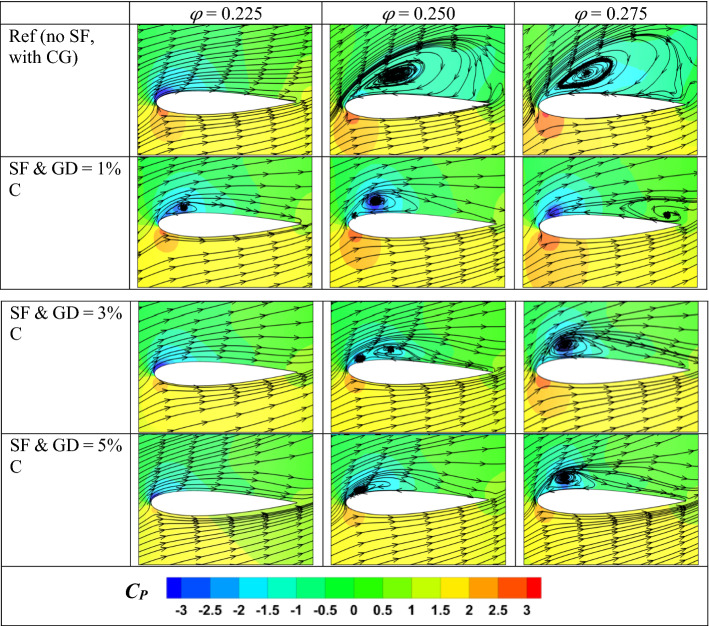
Pressure contours and streamlines near SF at 80% blade span.

A detailed analysis of flow is carried out in this section to understand the reason behind the change in performance for the modified turbine. The main area of interest for the flow analysis is the flow interaction between the CG and the SF at 80% blade span. Figure 10 shows the three flow coefficient pressure contours and streamlines near the 80% blade span. The pressure contours are represented in terms of static pressure coefficient defined as

6 $$C_{P} = \frac{{P_{i} - P_{a} }}{{\left( {1/2} \right)\rho u_{a}^{2} }}$$

where *P*_*a*_ is the pressure drop at the inlet and *P*_*i*_ is the pressure at any point in the computational domain. At *φ* = 0.225, the flow remains closely attached to the blade for reference and the modified turbines. However, a significant difference in streamlines can be visible at higher flow coefficients. The reference turbine stalls beyond *φ* = 0.225, visible from flow separation near the blade's leading edge at higher *φ*. However, for the turbines with fence and CG, the inception of flow separation occurs near the blade's leading edge but gets attached again close to the blade’s mid-chord. The fences here act as a vortex generator, which pulls fluid containing high energy from mainstream flow and mixes with the flow close to the boundary layer to initiate the flow reattachment. As a result, the stall point shifts towards the higher flow coefficient, although there is a loss of torque development due to some flow separation near the LE of the blade. Also, for the modified turbines, at a higher *φ*, the low-pressure region close to the SS of the blade is intense in a small area in contrast to the reference turbine, where it is spread towards the blade trailing edge direction.

**Figure 11:**
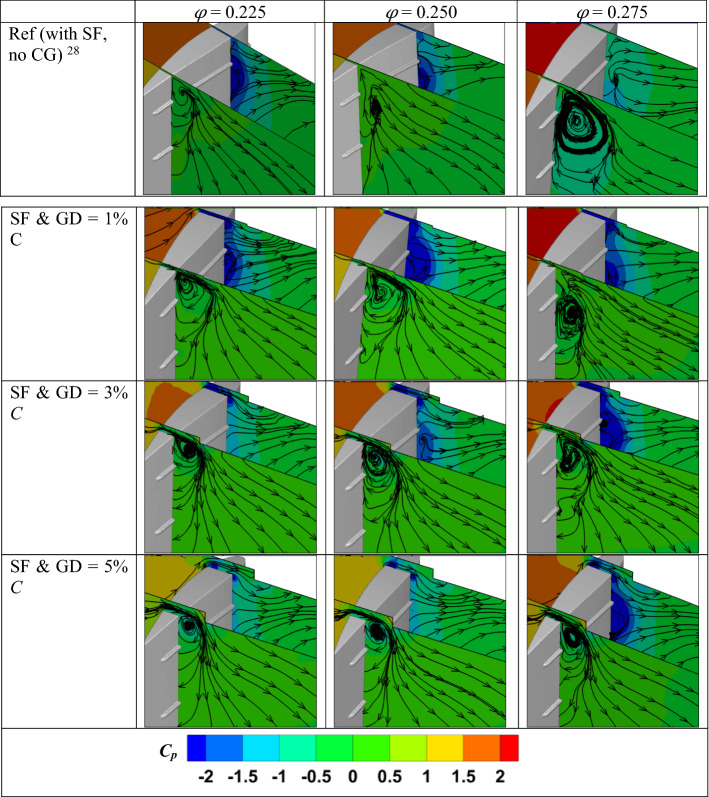
Streamline and pressure contours in different planes near the leading and trailing edges.

Figure 11 depicts the pressure contours and streamlines in two planes near the blade's leading and trailing edges. This will enable us to understand the effect of the CG and fence on the flow around the blade. In this case, the reference turbine is considered with SFs but without any CG. When no CG is used, the vortex forms close to the trailing edge due to the presence of the fence at 80% blade span. However, the tip leakage flow interacts with the mainstream flow when the CG is used. As a result, the vortex forms near the CG region, which tries to suppress the vortex near the fence region. Especially for 3 and 5% GD, the tip leakage vortex is strong enough to pull the fluids with high energy near the boundary layer, prohibiting further vortex formation from the blade. As a result, at *φ* = 0.275, the size of the vortex near the trailing edge is small when the CG is used compared to the reference case. This substantiates the wider stall point of the combined SF-CG turbine, as shown in Fig. 9. However, increasing the GD allows more tip leakage flow to pass through the tip region, which reduces the turbine performance.

**Figure 12:**
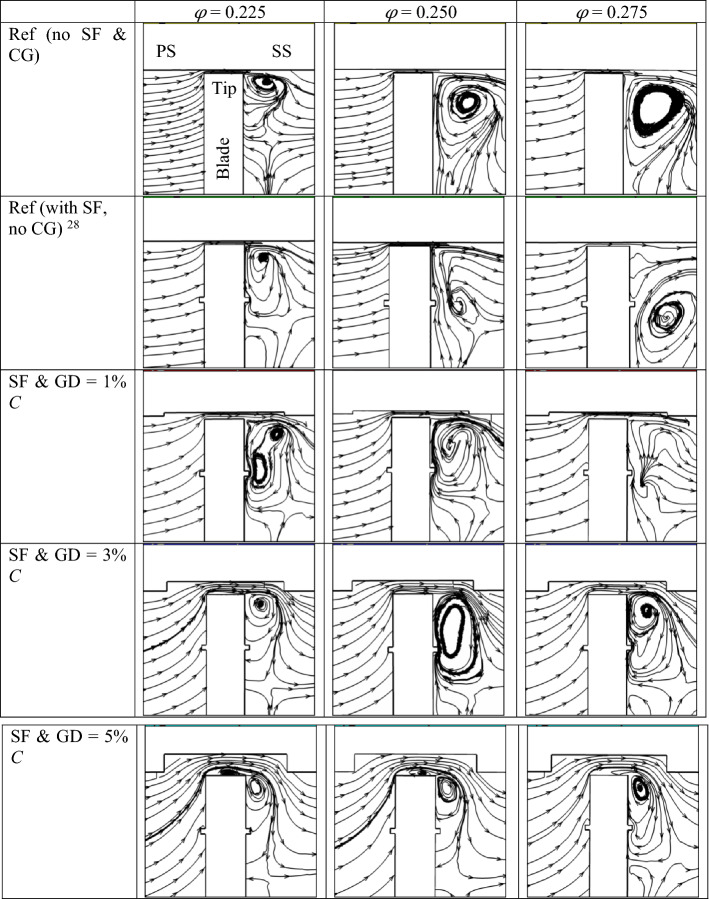
Streamlines at the mid chord of the blade show the fluid flow between the CG and top fence for different *φ*.

Figure 12 further analyzes the flow across the tip region and its interaction with the flow close to the top fence at a plane surface passing through the middle of the blade’s chord. A tip leakage vortex forms close to the tip zone when no fence or CG is used. The size of the vortex and strength increase with the increase in *φ*. When SFs are used, this tip leakage vortex interacts with the vortex formed due to the fences. As a result, the tip leakage vortex moves closer to the blade surface.

Further, some vortices are created inside the CG when a CG is used, which influences the vortex formation on the blade’s suction side (SS). However, this effect is not prominent with GD = 1% *C*. When GD is increased to 3 and 5% *C*, the vortex formed inside the CG pulls the high energy fluid close to the blade surface, resulting in a smaller size vortex on the SS of the blade. Also, the vortex moves up near the blade tip in contrast to the other cases where the vortex is formed down the blade tip close to the fence region. The fence and CG act as a passive flow controller to control the vortex formation on the blade’s suction side.

**Figure 13:**
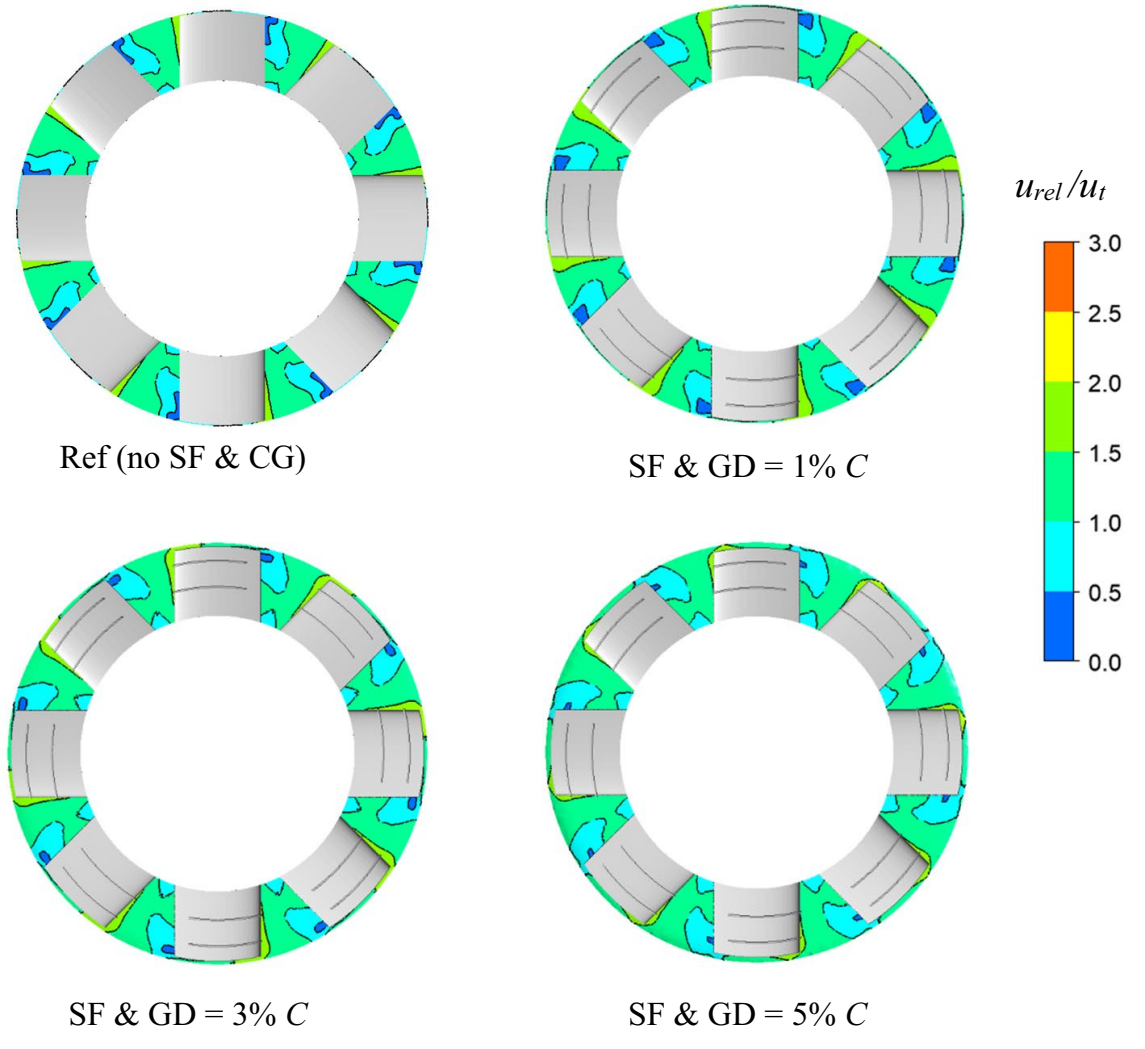
Comparison of velocity contours at 10% *C* from the blade surface on the suction side of the blade at *φ* = 0.275.

Figure 13 depicts the velocity contours at 10% *C* from the blade suction surface to better understand the flow behavior after passing through the blade at a high *φ* = 0.275. The relative velocity to tip velocity ratio is plotted as the velocity contours. In the case of an unaltered turbine, the low-velocity area extends from the tip to the midspan. The low-velocity region indicates vortex formation and flow separation leading to the stall phenomenon. As the GD increases, the low-velocity region becomes smaller and only close to the tip. This is because of the local vortex formation in the casing groove region, which suppresses the flow separation away from the blade.

## Conclusions

The present work discusses a modified blade geometry of Wells turbine combining stall fences and casing groove. Combining these two geometric modifications works as a passive flow control method to widen the turbine operating range. The CG, along with the fence, helps create a local vortex that pulls the high-energy fluid from mainstream flow to the boundary layer. As a result, flow reattachment occurs near the trailing edge at high flow coefficients, and subsequently, the turbine operating range increases. The operating range increases to 33.3% when the GD = 3% *C*. However, a further increase of GD increases the leakage flow causing a reduction in turbine performance. The increased operating range of the modified turbine comes at the expense of reduced torque production. However, the wide operating range will allow the turbine to work in varying inlet flow conditions compensating the power production. Furthermore, the turbine’s PTA power ratio decreased by 27.7%, allowing it to function smoothly over a wide range of flow coefficients. As the modified design requires more complex manufacturing than the reference turbine, it is recommended for future work to analyze the cost–benefit from the increased power compared to the manufacturing cost for the new design.

## Data Availability

The datasets used and/or analysed during the current study available from the corresponding author on reasonable request.

## List of symbols

$\Delta P_{0}$: Pressure drop across the rotor, Pa
$\Delta P_{0}^{*}$: Non-dimensional pressure drop
*C*: Chord length, mm
*C*_*p*_: Static pressure coefficient
*D*: The drag force, N
*F*_*N*_: The normal force, N
*F*_*T*_: Tangential force, N
*h*: Height of fence, mm
*l*: Fence length, mm
*L*: Lift (force), N
*p*: The span of the blade, mm
*P*_*i*_: Local static pressure, Pa
*P*_*a*_: Entrance static pressure, Pa
*Q*: Entrance volume flow rate, m^3^/s
*t*: Fence thickness, mm
*T*: Gross torque, N-m
*T**: Non-dimensional torque
*u*_*a*_: Entrance flow velocity, m/s
*u*_*t*_: Blade tip velocity, m/s
*u*_*rel*_: The relative velocity of fluid, m/s
*α*: The angle of incidence, degree
*η*: Efficiency
*ρ*: Air density, kg/m^3^
*φ*: Flow coefficient
*ω*_*r*_: Speed of rotation, rpm

## Abbreviations

AoA: Angle of attack
CG: Casing groove
GD: Groove depth
LE: Leading edge
OWC: Oscillating water column
PS: Pressure surface
PTA: Peak-to-average
PTO: Power take-off
SF: Stall fence
SS: Suction surface
SST: Shear stress transport
